# Gastric Cancer Screening Methods: A Comparative Study of the Chinese New Gastric Cancer Screening Score and Kyoto Classification of Gastritis

**DOI:** 10.1155/2022/7639968

**Published:** 2022-03-09

**Authors:** Xiao-ming Liu, Xiao-yu Ma, Fen Liu, Zhi-ling Liu, Xiang-yu Tang, Ming-zhu Ji, Jin-xin Zheng

**Affiliations:** ^1^Department of Gastroenterology, Shenzhen Qianhai Shekou Free Trade Zone Hospital, Shenzhen 518067, China; ^2^Department of Gastroenterology, Shenzhen Shekou People's Hospital, Shenzhen 518067, China; ^3^Department of Infectious Diseases and the Key Lab of Endogenous Infection, Huazhong University of Science and Technology Union Shenzhen Hospital, Shenzhen 518052, China; ^4^Department of Infectious Diseases and the Key Lab of Endogenous Infection, Shenzhen Nanshan People's Hospital and The 6th Affiliated Hospital of Shenzhen University Health Science Center, Shenzhen 518052, China

## Abstract

**Objective:**

To evaluate the Chinese new gastric cancer screening score (i.e., Li's score) and Kyoto Classification of Gastritis for screening gastric cancer.

**Methods:**

A total of 702 patients were scored using the two scoring methods. Gastric atrophy, intestinal metaplasia, and gastric cancer (including early gastric cancer) were compared between the two scoring methods. The area under the ROC curve, sensitivity, and specificity of the two scoring methods were evaluated.

**Results:**

Both of the two scoring methods found that gastric atrophy, intestinal metaplasia, and gastric cancer (including early gastric cancer) were all significantly higher in the medium-risk and high-risk group patients than those in the low-risk group patients. According to the Kyoto Classification of Gastritis, patients in the high-risk group had more gastric atrophy, intestinal metaplasia, and gastric cancer than those in the medium-risk group patients. Gastric atrophy, intestinal metaplasia, and gastric cancer in the low-risk and medium-risk group patients evaluated by the Li score were all significantly higher than those in patients with corresponding risk level evaluated by Kyoto Classification of Gastritis, respectively. The area under the ROC curve of the Li score was 0.702, and the sensitivity and specificity were 57.6% and 85.3%, respectively. The area under the ROC curve of the Kyoto Classification of Gastritis was 0.826, and the sensitivity and specificity were 75.4% and 83.6%, respectively.

**Conclusion:**

Both Li's score and Kyoto Classification of Gastritis showed good screening value for gastric cancer, but Kyoto Classification of Gastritis was more sensitive than the Li score.

## 1. Introduction

Gastric cancer is a common digestive tumor, threatening people's health, and is the focus of cancer prevention and treatment in the world [1]. The global distribution of morbidity and mortality associated with gastric cancer is greatly different and with the highest incidence in East Asia [2]. There were 679,000 new cases of gastric cancer and 498,000 deaths in China every year, accounting for 42.6% and 45.0% of the world, respectively. The diagnosis and treatment rate of early gastric cancer in China was less than 10%, much lower than that in Japan (70%) and South Korea (50%) [3–5]. The gastric cancer incidence in Guangdong (including Shenzhen) and Guangxi provinces was low [6]. Although great progress has been made in the diagnosis and treatment of gastric cancer, gastric cancer still has a high mortality. The 5-year survival rate of advanced gastric cancer was less than 30% in most countries [7]. However, the 5-year survival rate of early gastric cancer can be as high as 90% and even cured [8].

In East Asia, China and Japan both have high incidence rate of gastric cancer, but Japan has made great advances to better understanding the pathogenesis of gastric cancer and early endoscopic screening to reduce its prevalence [9]. Gastroscopy is an effective means to find early gastric cancer [10]. In 2013, the Japan Gastroenterological Endoscopy Society advocated the Kyoto Classification of Gastritis, a new grading system for endoscopic gastritis, to standardize the records of endoscopic manifestations of gastritis and evaluate the risk of gastric cancer [11]. Interestingly, recently, Japanese researchers found that Kyoto Classification of Gastritis can significantly improve the detection rate of early gastric cancer under gastroduodenal endoscopy [12].

The detection rate of early gastric cancer in China is less than 10%, far lower than that in Japan (70%) and South Korea (50%) [13]. Thus, the National Center for Clinical Medicine Research of Digestive Diseases (Shanghai) has recently conducted a multicenter clinical study, involving more than 100 hospitals in China, to establish a new gastric cancer screening scoring system, i.e., the Li-*Q* score [14]. The Chinese new gastric cancer screening scoring system (i.e., Li's score) was based on clinical and laboratory data but lacked the score of endoscopic manifestations. Conversely, the Kyoto Classification of Gastritis only based on the score of endoscopic manifestations but lacked the score of clinical and laboratory examinations. However, the two gastric cancer screening methods were compared and the accuracy of which was better is still unclear. Thus, this study was aimed at comparing the accuracy between the Chinese new gastric cancer screening score (i.e., Li's score) and Kyoto Classification of Gastritis, for gastric cancer prediction in the early cancer stages by screening patients with digestive system discomfort (outpatient or inpatient).

## 2. Patients and Methods

### 2.1. General Information

This study was a single-center, retrospective study. Patients (had digestive system symptoms such as abdominal pain, abdominal distention, nausea, vomiting, and hematemesis) who went to Shenzhen Qianhai Shekou Free Trade Zone Hospital and who had gastroscopy and biopsy completed during September 2019 to April 2021 were enrolled. The early gastric cancers were pathologically confirmed by endoscopic submucosal dissection (ESD), and advanced gastric cancers were pathologically determined by surgical specimens.

### 2.2. Patients

The patients included and excluded in this study were determined according to previous studies [15, 16]: (1) *inclusive criteria*: (1) age ≥ 40 years and (2) meet any of the following criteria: patients who lived in areas with high incidence of gastric cancer, or *Helicobacter pylori* infection, or precancerous gastric diseases such as chronic atrophic gastritis, gastric ulcer, gastric polyps, hypertrophic gastritis, and pernicious anemia, or first-degree relatives of patients with gastric cancer, or other risk factors for gastric cancer (such as high salt intake, pickled diet, smoking, and heavy drinking); (2) *Exclusive criteria* (meet any of the following criteria): (1) had severe cardiac, liver, and renal insufficiency, severe neuropathy, or mental disorders; (2) had gastric surgery history for gastric tumors (including surgery, ESD, and endoscopic mucosal resection (EMR)); and (3) had a tendency to bleed and cannot undergo a biopsy (platelets less than 50 or acute upper gastrointestinal bleeding).

This study has been approved by the Ethics Committee of Shenzhen Qianhai Shekou Free Trade Zone Hospital, and the consent of patients has been obtained. All patients were informed about the purpose of the study and that it was conducted in accordance with the Declaration of Helsinki.

### 2.3. Gastroscopic and Histological Examination

In addition to white light gastroscopy, any further available examinations including narrow-band imaging, magnifying endoscopy, and chromoendoscopy were conducted by Olympus 290 series. Gastroscopy was performed by expert endoscopists who had performed endoscopy examination or therapy for more than 1000 cases.

Gastric biopsy specimens were obtained from gastroesophageal junction, gastric body, gastric angle, and gastric antrum. Each specimen was independently reviewed by two pathologists. Gastric inflammation and atrophy were determined according to the Updated Sydney System [17]. Gastric cancers were diagnosed by the criteria from the Vienna classification for gastrointestinal epithelial neoplasia [18].

### 2.4. Chinese New Gastric Cancer Screening Scoring System

The new gastric cancer screening scoring system has been established in the expert consensus opinion of the early gastric cancer screening project in China [16, 19]. The Chinese new gastric cancer screening scoring system include 5 variables with a total score of 23: (1) age: 0 for 40-49, 5 for 50-59, 6 for 60-69, and 10 for >69; (2) sex: 4 for male and 0 for female; (3) *H. pylori* infection (Hp antibody test): positive 1 and negative 0; (4) PGR (serum pepsinogen I/II ratio): ≥3.89 is 0 and <3.89 is 3; and (5) G-17 (gastrin 17): less than 1.50 is 0, 1.50-5.70 is 3, and greater than 5.70 is 5 (Table 1). Gastric cancer risk stratification according to total scores is as follows: 0-11 regarded as low risk, 12-16 regarded as medium risk, and 17-23 regarded as high risk.

### 2.5. Kyoto Classification of Gastritis

The Kyoto Classification of Gastritis was advocated by the 85^th^ Congress of the Japan Gastroenterological Endoscopy Society in 2013 [13]. The Kyoto Classification of Gastritis was based on the sum of scores of the five endoscopic findings (atrophy, intestinal metaplasia, enlarged folds (tortuous folds), nodularity, and diffuse redness) and ranges from 0 to 8 (Table 2). According to previous research [13], gastric cancer risk stratification according to Kyoto Classification of Gastritis scoring system is as follows: scores < 2 regarded as low risk, 2 ≤ scores < 4 regarded as medium risk, and scores ≥ 4 regarded as high risk.

### 2.6. Statistical Analysis

All the data was analyzed by SPSS ver. 19.0 software. Categorized variables were expressed by percentage (%) and analyzed by the chi-square test. ROC curve was used to determine the sensitivity and specificity. *P* < 0.05 was statistically significant.

## 3. Results

### 3.1. General Situation

A total of 702 patients were included in this study, 366 males and 336 females, aged 40-85 years (median: 56 years). Gastric cancer risk stratification in 702 patients according to the Chinese new gastric cancer screening score (i.e., Li's score) is as follows: 585 low-risk cases (83.33%), 93 medium-risk cases (13.25%) and 24 high-risk cases (3.42%), while according to the Kyoto Classification of Gastritis, it is as follows: 384 low-risk cases (54.70%), 171 medium-risk cases (24.36%), and 147 high-risk cases (20.94%).

### 3.2. *Helicobacter pylori* Infection according to the Kyoto Classification of Gastritis

There were 33 (8.59%), 105 (61.40%), and 126 (85.71%) *H. pylori* infection cases found in the low-risk, medium-risk, and high-risk group patients, respectively. The *H. pylori* infection rate in the medium-risk and high-risk group patients was significantly higher than that in the low-risk group patients. The high-risk group patients also had a higher *H. pylori* infection rate than the medium-risk group patients (Table 3).

Two cases of gastric cancer were found in the low-risk group patients, all of which were advanced gastric cancer, but *H. pylori* was negative. In the medium-risk group patients, 10 cases of gastric cancer were found, and 7 cases (70.00%) were *H. pylori* positive. Among those 10 cases of gastric cancer (medium-risk group patients), there were 3 cases of early gastric cancer: 2 cases of *H. pylori* positive and 1 case after *H. pylori* eradication therapy. Among the high-risk group patients, 28 cases of gastric cancer were found, and 23 cases were *H. pylori* positive, including 3 cases of early gastric cancer (Table 3).

### 3.3. Gastric Atrophy and Intestinal Metaplasia Were Evaluated by the Two Scoring Methods

According to the Chinese new gastric cancer screening score, gastric atrophy and intestinal metaplasia in the medium-risk and high-risk group patients were all significantly higher than those in the low-risk group patients. Similar to the above results, Kyoto Classification of Gastritis also found that patients in the medium-risk and high-risk groups had higher positive rates of atrophy and intestinal metaplasia than those in the low-risk group patients. Moreover, patients in the high-risk group had more gastric atrophy and intestinal metaplasia than those in the medium-risk group patients, which were evaluated by Kyoto Classification of Gastritis (Table 4). Interestingly, as Table 4 also indicated, gastric atrophy and intestinal metaplasia in the low-risk and medium-risk group patients, which were evaluated by the Chinese new gastric cancer screening score, were all significantly higher than those in patients with corresponding risk level evaluated by Kyoto Classification of Gastritis, respectively.

### 3.4. Comparison of Gastric Cancer Results between the Two Scoring Methods

Gastric cancer was detected in 40 of 702 (5.70%) patients in this study, including early gastric cancer 6 cases (high-grade intraepithelial neoplasia 2 cases and mucosal cancer 4 cases). As Table 5 indicated, both of the two scoring methods found that gastric cancer and early gastric cancer were more common in the medium-risk and high-risk group patients than those in the low-risk group patients. In addition, patients in the high-risk group had more gastric cancer than those in the medium-risk group, which were assessed by Kyoto Classification of Gastritis. According to the Chinese new gastric cancer screening score, the low-risk and medium-risk group patients had more gastric cancer than in patients with corresponding risk level evaluated by Kyoto Classification of Gastritis (Table 5).

The histologic type (differentiated and undifferentiated) and location (gastroesophageal junction, gastric body, gastric angle, and gastric antrum) of 40 cases of gastric cancer were also analyzed in this study (Table 6). However, due to the small number of gastric cancer cases, statistical analysis was not carried out.

### 3.5. Comparison of Correlation and Accuracy between the Two Scoring Methods

The correlation between the two scoring methods was analyzed by the *kappa* test, and the results showed that there was no correlation between them: *κ* = 0.145, *P* < 0.001.

**Figure 1:**
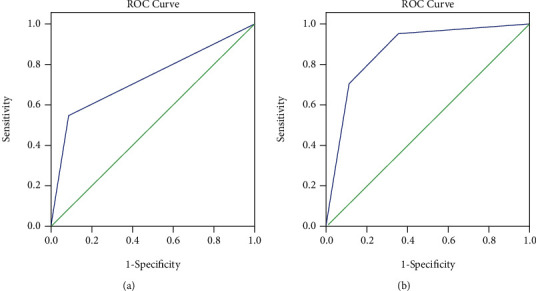
ROC curve analysis of two scoring methods to diagnose gastric cancer. (a) ROC curve analysis of Chinese new gastric cancer screening score (i.e., Li's score). (b) ROC curve analysis of Kyoto Classification of Gastritis.

The area under the ROC curve of the Chinese new gastric cancer screening score of gastric cancer was 0.702, and the sensitivity and specificity were 57.6% and 85.3%, respectively. However, the area under the ROC curve of the Kyoto Classification of Gastritis was 0.826, and the sensitivity and specificity were 75.4% and 83.6%, respectively (Figure 1).

## 4. Discussion

Gastric cancer is one of the common malignant tumors. The early detection, diagnosis, and treatment of gastric cancer play an important role in improving the survival rate of gastric cancer and reducing medical expenditure [19]. At present, China still faces the situation of large population and uneven distribution of medical resources, so the feasibility of recommending all patients to carry out gastroscope screening for gastric cancer is relatively poor. Therefore, it is urgent to explore an efficient and feasible alternative to gastroscopy for gastric cancer screening.

China is a high infection area of *H. pylori* in the world, and *H. pylori* infection has been identified as the main cause of gastric cancer [20]. Thus, a total of 5 variables, age, gender difference, *H. pylori* antibody, PGR, and G-17, were assigned different scores in the Chinese new gastric cancer screening score (i.e., Li's score). Based on the Chinese new gastric cancer screening score, this study confirmed that gastric atrophy, intestinal metaplasia, and gastric cancer in the medium-risk and high-risk group patients were significantly higher than those in the low-risk group patients. These results suggest that the Chinese new gastric cancer screening score can improve the detection rate of precancerous lesions and gastric cancer in Chinese patients. However, the present study also found that the Chinese new gastric cancer screening score could not improve the detection rate of precancerous lesions and gastric cancer between the high-risk and medium-risk group patients. What is more, the area under the ROC curve, the sensitivity, and specificity of the Chinese new gastric cancer screening score were all poor than those of the Kyoto Classification of Gastritis.

Based on the difference results of the above two gastric cancer screening scoring methods, we believe that the Chinese new gastric cancer screening score is not suitable for screening precancerous lesions and gastric cancer, but it is suitable for general screening of the population in developing countries like China and further formulating follow-up plans. It is identified as the medium-risk and high-risk patients by the Chinese new gastric cancer screening score and then accepts gastroscope screening (according to the Kyoto Classification of Gastritis) to find out the real high-risk patients of gastric cancer, which can save human and material resources and is more in line with the needs of developing countries such as China.

It is well known that *H. pylori* infection, gastric atrophy, and intestinal metaplasia are closely related to the occurrence of gastric cancer [21]. The results of this study suggested that Kyoto Classification of Gastritis was helpful to find gastric precancerous lesions. The early gastric cancer in the high-risk group was significantly higher than that in the low-risk group, suggesting that patients with a score of ≥4 (according to Kyoto Classification of Gastritis) were examined by a magnifying gastroscope which would be helpful to improve the detection rate of early gastric cancer. Based on the above results, gastroscopy is recommended for patients with high scores (Kyoto Classification of Gastritis ≥ 4), which may improve the detection rate of early gastric cancer, improve the survival rate, reduce the mortality, and save medical expenses.

This study was limited by its single-center and retrospective nature and small number of gastric cancer cases. Moreover, we know that Kyoto gastritis classification tends to detect intestinal gastric cancer related to Helicobacter pylori infection, and the positive rate of gastric cancer under the background of Helicobacter pylori-negative gastric mucosa is not good. Thus, to some extent, it may affect the accuracy and reliability of the results. Therefore, the above screening scheme may lead to poor detection ability of this part of gastric cancer. Therefore, further classification methods for detecting gastric cancer without Helicobacter pylori infection need to be studied.

## 5. Conclusion

The present study demonstrated that both Chinese new gastric cancer screening score and Kyoto Classification of Gastritis showed good screening value for gastric cancer in patients, but Kyoto Classification of Gastritis was more sensitive than the Chinese new gastric cancer screening score. The Chinese new gastric cancer screening score is suitable for general screening of the population in developing countries like China, and the medium-risk and high-risk patients identified by the Chinese new gastric cancer screening score are suitable for the gastroscope screening (according to the Kyoto Classification of Gastritis) to find out the real high-risk patients of gastric cancer.

## Figures and Tables

**Table 1:** Chinese new gastric cancer screening scoring system.

Variates and classification	Score
Age (years)	
40-49	0
50-59	5
60-69	6
>69	10
Gender	
Female	0
Male	4
*H. pylori* antibody	
Negative	0
Positive	1
Serum pepsinogen I/II ratio	
≥3.89	0
<3.89	3
Gastrin 17 (pmol/L)	
<1.50	0
1.50-5.70	3
>5.70	5

The Chinese new gastric cancer screening scoring system include 5 variables with a total score of 23. Gastric cancer risk stratification according to total scores: 0-11 regarded as low risk, 12-16 regarded as medium risk, and 17-23 regarded as high risk.

**Table 2:** Kyoto Classification of Gastritis scoring system.

Variates and classification	Score
Gastric mucosal atrophy	
C0–CI	0
CII–CIII	1
OI–OIII	2
Intestinal metaplasia	
None	0
Within the antrum	1
Up to the corpus	2
Hypertrophy of gastric fold	
<5 mm gastric fold width	0
≥5 mm gastric fold width	1
Nodularity	
None	0
Small nodules in the antrum	1
Diffuse redness	
None	0
Mild translucency of collecting venules in the body	1
Severe translucency of collecting venules in the body	2

The Kyoto Classification of Gastritis was based on the sum of scores of the five endoscopic findings and ranges from 0 to 8. According to previous research [8], gastric cancer risk stratification according to Kyoto Classification of Gastritis scoring system is as follows: scores < 2 regarded as low risk, 2 ≤ scores < 4 regarded as medium risk, and scores ≥ 4 regarded as high risk.

**Table 3:** *Helicobacter pylori* infection according to the Kyoto Classification of Gastritis.

Risk stratification	No.	*H. pylori* infection	*P* values	Gastric cancer		
				No.	*H. pylori+*	*H. pylori-*
Low risk	384	33 (8.59%)	<0.001^a^	2	0	2 (100%)
Medium risk	171	105 (61.40%)	<0.001^b^	10	7 (70%)	3 (30%)
High risk	147	126 (85.71%)	<0.001^c^	28	23 (82.14%)	5 (17.86%)

*H. pylori* infection: ^a^low-risk group vs. medium-risk group; ^b^medium-risk group vs. high-risk group; ^c^high-risk group vs. low-risk group; +: positive; -: negative; No.: case number.

**Table 4:** Comparison of gastric atrophy and intestinal metaplasia results between the two scoring methods.

Scoring method/risk stratification	Gastric atrophy	*P* values	Intestinal metaplasia	*P* values
Chinese new gastric cancer screening scoring system (Li's score)				
Low risk (No. 585)	156 (26.67%)^¶^	<0.001^a^	129 (22.05%)^¶^	<0.001^a^
Medium risk (No. 93)	75 (80.65%)^★^		75 (80.65%)^★^	
High risk (No. 24)	21 (88.89%)	*<*0.001^c^	21 (88.89%)	*<*0.001*^c^*
Kyoto classification of gastritis scoring system				
Low risk (No. 384)	27 (7.03%)	<0.001^a^	18 (4.69%)	<0.001^a^
Medium risk (No. 171)	78 (45.61%)	<0.001^b^	66 (38.60%)	*<*0.001^b^
High risk (No. 147)	147 (100%)	<0.001^c^	141 (95.92%)	*<*0.001^c^

^a^Low-risk group vs. medium-risk group; ^b^medium-risk group vs. high-risk group; ^c^high-risk group vs. low-risk group; ^¶^low-risk group: Chinese new gastric cancer screening scoring system vs. Kyoto Classification of Gastritis scoring system, *P* < 0.001; ^★^medium-risk group: Chinese new gastric cancer screening scoring system vs. Kyoto Classification of Gastritis scoring system, *P* < 0.001. No.: case number.

**Table 5:** Comparison of gastric cancer results between the two scoring methods.

Scoring method/risk stratification	GC	*P* values	Early GC	*P* values
Chinese new gastric cancer screening scoring system (Li's score)				
Low risk (No. 585)	18 (3.08%)^¶^	<0.001^a^	2 (0.34%)	<0.05^a^
Medium risk (No. 93)	18 (19.35%)^★^		3 (3.23%)	
High risk (No. 24)	4 (16.67%)	<0.05^c^	1 (4.17%)	*<*0.001*^c^*
Kyoto classification of gastritis scoring system				
Low risk (No. 384)	2 (0.52%)	<0.001^a^	0	<0.05^a^
Medium risk (No. 171)	10 (5.85%)	<0.001^b^	3 (1.75%)	
High risk (No. 147)	28 (19.05%)	<0.001^c^	3 (2.04%)	*<*0.05^c^

^a^Low-risk group vs. medium-risk group; ^b^medium-risk group vs. high-risk group; ^c^high-risk group vs. low-risk group; ^¶^low-risk group: Chinese new gastric cancer screening scoring system vs. Kyoto Classification of Gastritis scoring system, *P* = 0.006; ^★^medium-risk group: Chinese new gastric cancer screening scoring system vs. Kyoto Classification of Gastritis scoring system, *P* = 0.001. No.: case number; GC: gastric cancer.

**Table 6:** Comparison of histologic type and location of gastric cancer results between the two scoring methods.

Scoring method/risk stratification	Histologic type of GC		Location of GC			
	Differentiated	Undifferentiated	GEJ	Gastric body	Gastric angle	Gastric antrum
Chinese new gastric cancer screening scoring system (Li's score)						
Low risk (No. 585)	4 (0.68%)	14 (2.39%)	4 (0.68%)	1 (0.17%)	2 (0.34%)	11 (1.88%)
Medium risk (No. 93)	5 (5.38%)	13 (13.98%)	3 (3.23%)	2 (2.15%)	2 (2.15%)	11 (11.83%)
High risk (No. 24)	1 (4.17%)	3 (12.50%)	0	1 (4.17%)	1 (4.17%)	2 (8.33%)
Kyoto Classification of Gastritis scoring system						
Low risk (No. 384)	1 (0.26%)	1 (0.26%)	0	2 (0.52%)	0	0
Medium risk (No. 171)	3 (1.75%)	7 (4.09%)	2 (1.17%)	0	2 (1.17%)	6 (3.51%)
High risk (No. 147)	6 (4.08%)	22 (14.97%)	5 (3.40%)	2 (1.36%)	3 (2.04%)	18 (12.24%)

No.: case number; GC: gastric cancer; GEJ: gastroesophageal junction.

## Data Availability

The patients' data used to support the findings of this study are available from the corresponding author upon request.
